# Supplementary material for: ScATG8 Gene Cloned from Desert Moss Syntrichia caninervis Exhibits Multiple Stress Tolerance
Source: Plants (Basel). 2023 Dec 23;13(1):59. doi: 10.3390/plants13010059 (PMC10780840; doi:10.3390/plants13010059)
Supplement: Supplementary file 1 [file plants-13-00059-s001.zip › supplemental figures and legends.pdf]

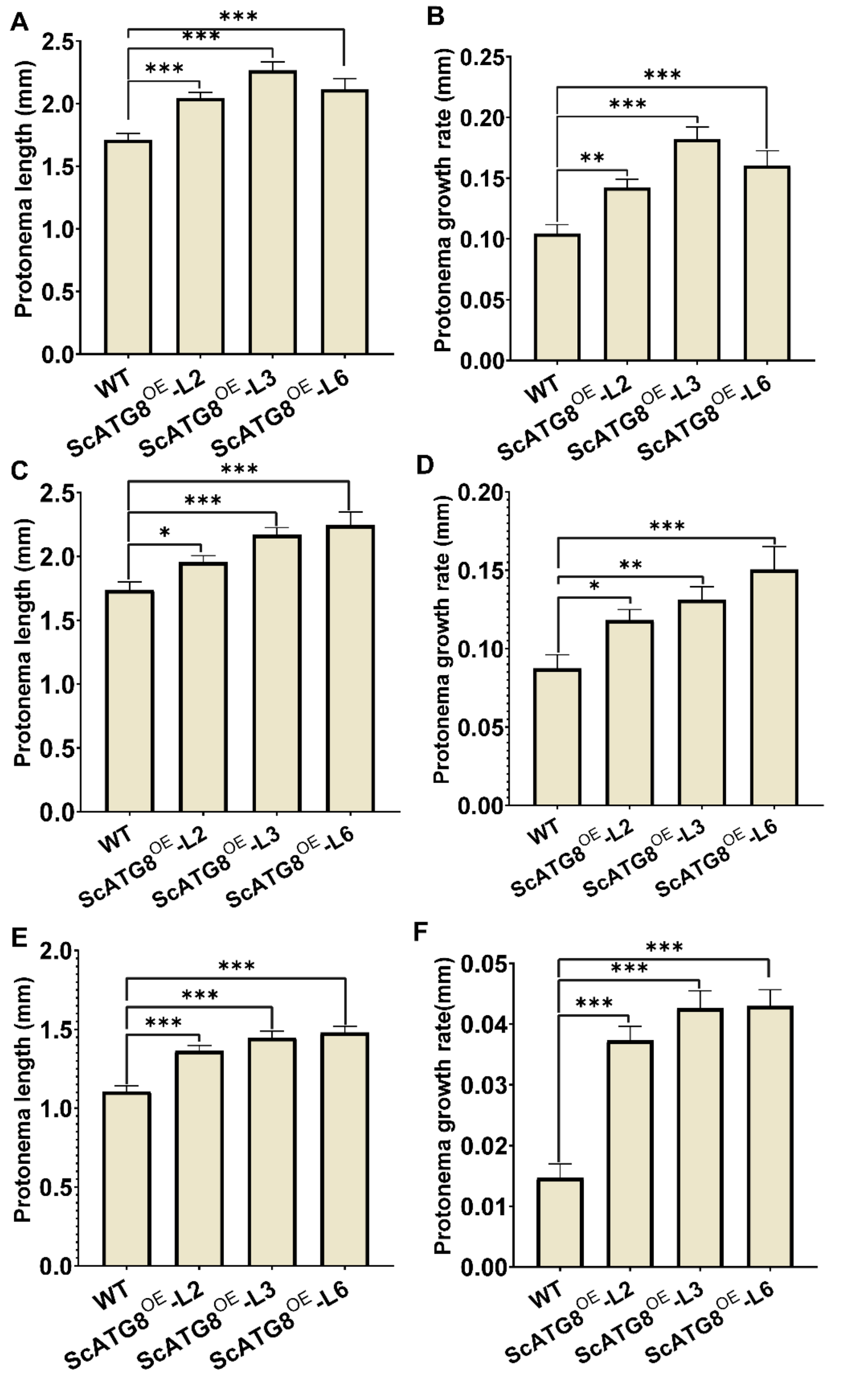

Figure S1. Growth rate of *ScATG8* transgenic *P. patens* under different stress conditions. Protonemal length of ScATG8^{OE} transgenic *P. patens* under cold(A), heat(C), and salt(E). Protonema growth rate of ScATG8^{OE} transgenic *P. patens* under cold(B), heat(D), and salt(F). For each treatment, total of 20 colonies were used. Values were presented means of n = 20 ± SD, significance was determined by the ANOVA (**P* < 0.05, ***P* < 0.01, ****P* < 0.001).
